# The effect of decellularisation on the real time mechanical fatigue of porcine aortic heart valve roots

**DOI:** 10.1371/journal.pone.0265763

**Published:** 2022-04-01

**Authors:** Amisha Desai, Eileen Ingham, Helen E. Berry, John Fisher, Louise M. Jennings

**Affiliations:** 1 Institute of Medical & Biological Engineering, School of Mechanical Engineering, University of Leeds, Leeds, United Kingdom; 2 Institute of Medical & Biological Engineering, Faculty of Biological Sciences, University of Leeds, Leeds, United Kingdom; Politecnico di Milano, ITALY

## Abstract

Decellularised heart valve roots offer a promising option for heart valve replacement in young patients, having the potential to remodel and repair. Replacement heart valves have to undergo billions of opening and closing cycles throughout the patient’s lifetime. Therefore, understanding the effect of cyclic loading on decellularised heart valve roots is important prior to human implantation. The aim of this preliminary study was to investigate the influence of low concentration sodium dodecyl sulphate (SDS) decellularisation treatment on the *in vitro* real time mechanical fatigue of porcine aortic heart valve roots under physiological real time cyclic loading conditions. This required a specific real time *in vitro* method to be developed, since previous methods relied on accelerated testing, which is non-physiological, and not appropriate for valve replacement materials that exhibit time dependent characteristics. The effects of the real time fatigue on hydrodynamic function and mechanical properties of the heart valve roots were assessed. The mechanical fatigue of decellularised porcine aortic heart valve roots (n = 6) was assessed and compared to cellular porcine aortic heart valve roots (n = 6) in a modified Real time Wear Tester (RWT) at a physiological frequency and under cyclic pressure conditions for a maximum of 1.2 million cycles. Periodically, the heart valve roots were removed from the RWT to assess the influence of cyclic loading on valve competency (static leaflet closure). At the end of testing further hydrodynamic performance parameters were ascertained, along with determination of leaflet material properties. A real time mechanical fatigue assessment method was developed and applied; with two cellular and two decellularised porcine aortic leaflets in different heart valve roots showing tears in the belly region. The decellularised aortic heart valve roots exhibited comparative functionality to the cellular heart valve roots under *in vitro* static and pulsatile hydrodynamic conditions. However, the material properties of the decellularised aortic leaflets were significantly altered following cyclic fatigue assessment and showed increases in elastin and collagen phase slopes and ultimate tensile strength compared to the cellular porcine aortic leaflets in the circumferential direction. This preliminary study demonstrated that low concentration SDS decellularised porcine aortic heart valve roots can withstand physiological cyclic deformations up to 1.2 million cycles in a RWT whilst maintaining their overall hydrodynamic function and leaflet mechanical properties. This is the first full report of preclinical mechanical fatigue assessment of decellularised porcine aortic heart valve roots under physiological real time conditions.

## Introduction

Valvular heart valve disease is a major cause of morbidity and mortality worldwide. One of the solutions to this problem has been heart valve replacement, which is currently performed over 300,000 times each year worldwide [1]. Whilst current replacement heart valve options can provide an effective treatment for many patients, limitations such as risk of thromboembolism with mechanical valves, valvular degeneration with bioprosthetic valves, absence of regeneration and growth in both mechanical and bioprosthetic valves and root dilation and regurgitation with allografts and autografts can lead to problems especially when implanted in young patients. To overcome these problems, many research groups have investigated the potential of decellularised heart valves.

Indeed, in recent years, decellularised human allograft heart valve roots have shown promising results in clinical studies [2–8]. The decellularisation process maintains the structure of the tissue therefore the valves have excellent hemodynamics and the potential repopulation with the recipients endogenous cells into the valve gives the tissue the capacity to grow, repair and remodel. Decellularised human allograft heart valve roots are however, limited in the availability of sizes appropriate for young patients. To address this problem, “off the shelf” decellularised porcine aortic and pulmonary heart valve roots have been investigated by several groups [9–12]. Initial clinical translation of the SynerGraft® decellularised porcine heart valve roots proved to be very poor [13, 14]. Subsequent findings reported incomplete decellularisation; this resulted in severe inflammation and a foreign body reaction, which was one of the main reasons for failure of the SynerGraft® decellularised porcine heart valve roots.

The clinical success of decellularised xenogeneic heart valves will be highly reliant upon the ability of the decellularisation process to remove all the cellular components in order to avoid an adverse immune response following implantation. It was determined that 0.1% (w/v) sodium dodecyl sulphate (SDS) facilitated the removal of cellular material without causing substantial damage to the extracellular matrix [15] and this concentration was subsequently adopted in the Leeds proprietary decellularisation protocol for the development of decellularised porcine [11, 9] and human [16] pulmonary and aortic heart valve roots.

*In vivo*, heart valves are subjected to a unique combination of mechanical factors; bending deformation of the leaflets that results in shear, compression and tension internal stresses. These complex mechanical factors can lead to mechanical failure [17, 18]. Most studies on the mechanical response of biological heart valves have focused on the investigation of functional properties [uniaxial or biaxial tensile quasi-static mechanical properties [9, 19–21] and flexural properties [22, 23] however, the fatigue of heart valve roots under physiological conditions is still not well understood. *In vitro* fatigue assessment studies [24–27] investigating the durability of mechanical and bioprosthetic heart valve prostheses have been widely reported in literature. However, such studies have used accelerated fatigue testers at significantly higher frequencies (15 to 20 Hz) than physiological frequencies (1 to 3 Hz), in order to predict fatigue from a high number of cycles in a reasonable time period. The major limitation of the accelerated fatigue assessment method is that valve leaflet motion (valve leaflet opening and closing) is non-physiological, and not appropriate for valve replacement materials that exhibit time dependent characteristics. D’Souza, Butterfield [25] further explained that the leaflet kinematics, bending strains in the leaflet belly, the strain time, integral and proportional of time that the valve fully closed or opened were dependent on cycle rate. Furthermore the time dependent material properties of decellularised heart valve roots and polymer heart valves are quite different to xenogeneic cross linked heart valve tissue, and unlike cross linked tissue, the acceleration of fatigue and durability tests for decellularised valves have not yet been investigated or validated. Hence, these accelerated fatigue assessment methods are not, at the moment, appropriate for use with biological heart valve roots such as decellularised heart valve roots, which exhibit highly time dependent viscoelastic behaviour.

*In vitro* fatigue assessment studies [28–30] investigating the fatigue properties of frame mounted tissue valves with viscoelastic properties have been reported in literature. However, the fatigue test methods used in these studies were not suitable for decellularised heart valve roots as visco-elastic effects of the aorta were not included. More commonly, the fatigue of decellularised heart valve roots has been investigated in the *in vivo* juvenile sheep model [31–35]. Currently, there are no published studies reporting the development of an *in vitro* real time fatigue assessment method, capable of the preclinical mechanical fatigue assessment of decellularised heart valve roots under physiological conditions. Furthermore there have been no *in vitro* studies of the effect of acceleration of fatigue testing on these valve types. The aim of this study was to investigate the influence of 0.1% (w/v) SDS decellularisation on the mechanical fatigue of porcine aortic heart valve roots. This required a specific *in vitro* method to be developed. Post-fatigue analysis of the hydrodynamic function and mechanical properties of the heart valve roots was performed.

## Materials, study design and methods

### Cellular and decellularised porcine aortic heart valve roots

Aortic heart valve roots were obtained from 24–28 week old pigs, supplied within 5–7 hours of slaughter from two local abattoirs supplying the food chain. The internal diameter of each heart valve root was measured using cylindrical sizing instruments. Heart valve roots were allocated into two groups for tissue processing: decellularised porcine aortic heart valve roots (n = 6; D1 to D6) and cellular porcine aortic heart valve roots (n = 6; C1 to C6). The decellularised porcine aortic heart valve roots were processed using a proprietary low concentration SDS based protocol [36] and stored at 4° C in 150 ml phosphate buffered saline (PBS) until testing; for the cellular aortic heart valve roots, arteries of the heart valve roots were ligated and were submersed in treatment solution [150 mL Cambridge antibiotic (Source BioScience; 80 rpm) + 2.5 mL amphotericin B (Sigma-Aldrich Company Ltd.)] for 3 hours at 37°C temperature using an Optima™ T 100 heated circulating bath to minimise growth of contaminating bacteria and fungi in the real time wear tester. All the treated cellular porcine aortic heart valve roots were stored in 150 ml treatment solution and used immediately for testing.

#### Study design

**Fig 1:**
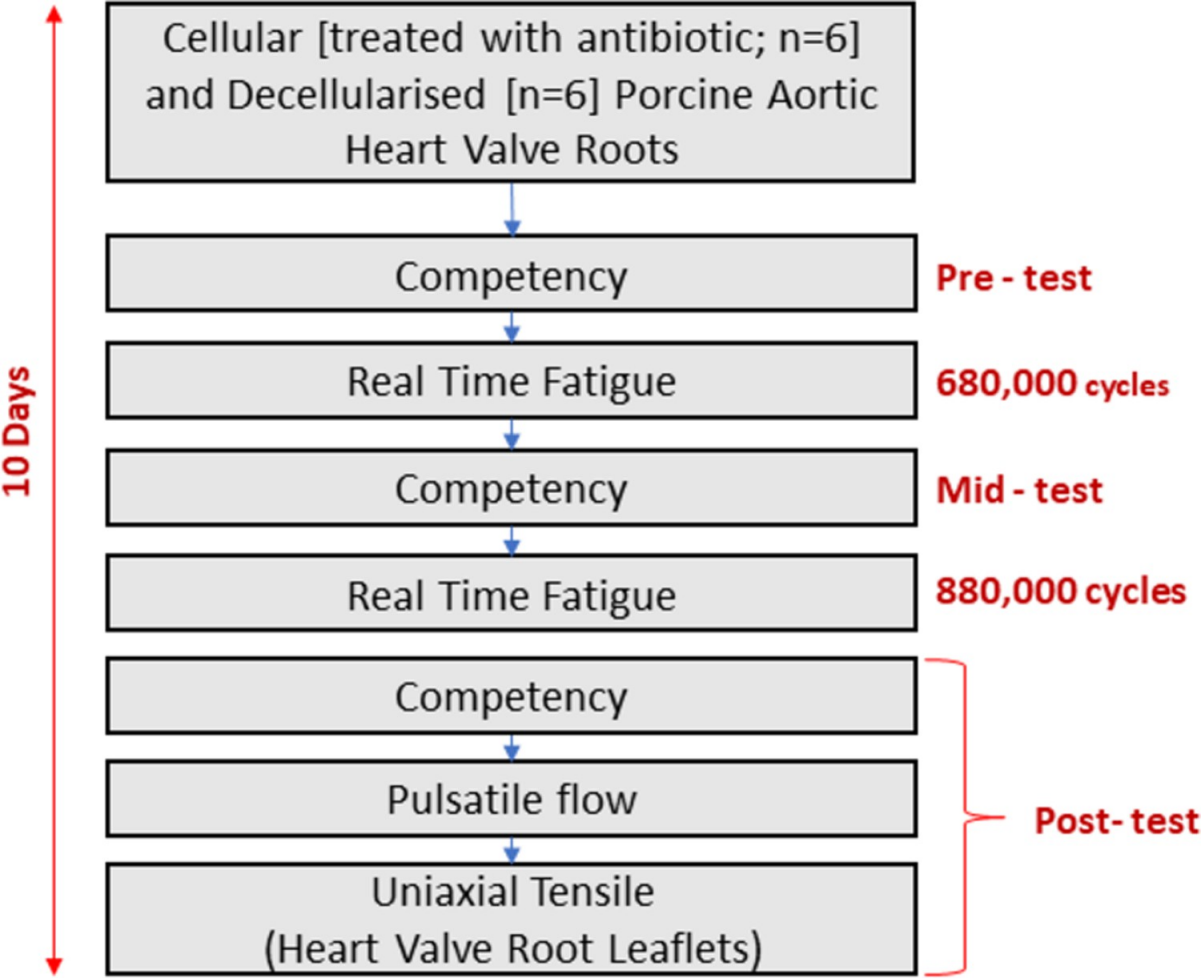
Flow chart indicating the sequence of biomechanical performance assessment of cellular and decellularised porcine aortic heart valve roots.

The *in vitro* real time mechanical fatigue of the porcine aortic cellular and decellularised heart valve roots was investigated in sets of n = 3 (four studies) under physiological cyclic loading conditions in a modified real time wear tester for up to 1.2 million cycles. To quantitatively determine the effect of cyclic loading on the heart valve roots, the hydrodynamic (competency/leakage and/or pulsatile flow) and biomechanical (uniaxial tensile properties of leaflets) performance was assessed at pre-defined intervals as shown in Fig 1.

Biological assessment showing effective decellularisation of porcine aortic heart valve roots using 0.1% (w/v) SDS (Leeds proprietary protocol) has previously been reported [11, 15], and was outside the scope of this study.

#### Description of the real time wear tester

**Fig 2:**
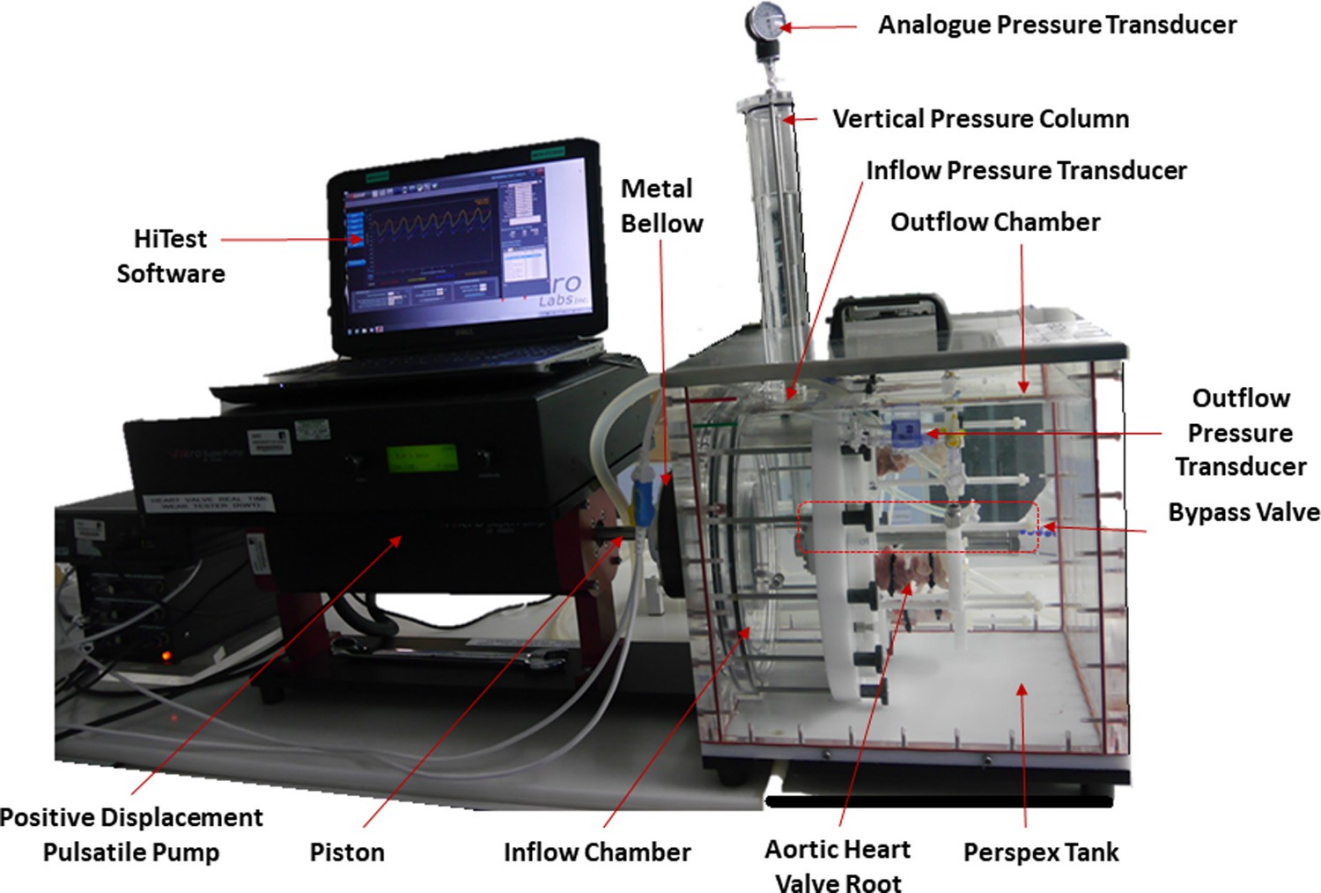
Image of the modified real time wear tester.

**Fig 3:**
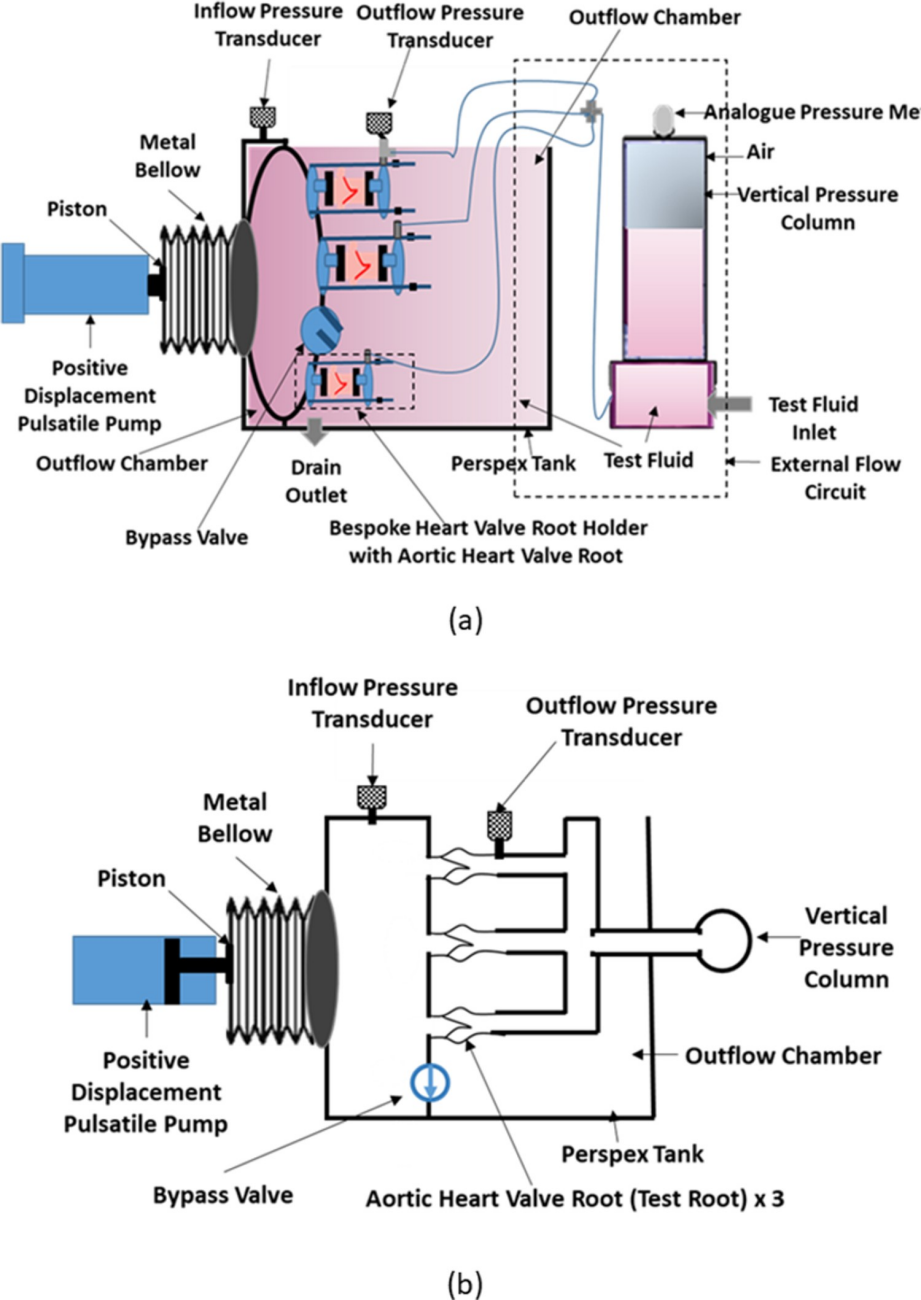
(a) The schematic diagram of the modified real time wear tester with position of inflow and outflow pressure transducers (b) simplified schematic of the modified real time wear tester.

The real time wear tester [RWT; Vivitro Systems Inc, Victoria BC, Canada (Fig 2) consisted of six equally spaced heart valve mounting stations in acrylic chambers, integrated between a closed inflow and opened outflow chamber housed in a Perspex tank. The RWT was attached to a digitally controlled microprocessor based pumping system which was capable of recreating *in vivo* pulsatile flow input waveforms with physiological heart rates 60, 120 and 200 bpm. In terms of function, when the pump piston travelled backwards (moved away from the test valve), the metal bellow expanded, and a volume of test solution was drawn into the inflow chamber through the test valve hence closing the valve leaflets. On the forward piston stroke, the test solution was expelled, the metal bellow compressed, opening the test valve. There was no flow meter in the system. A digital pressure transducer attached on the top of the inflow chamber, and therefore aligned with the inflow side of the test heart valves, measured back pressure. The inflow pressure was recorded as a negative value due to the movement of the bellow resulting in suction pressure. In the original unmodified configuration, another digital transducer, open to the outflow chamber, measured outflow pressure. The pressure across the closed valve was controlled using a one-way adjustable mechanical bypass valve located between the inflow and outflow chamber (Fig 3), and by regulating the amplitude of the input waveforms. Three mono leaflet mechanical heart valves were used to evaluate the function of the RWT prior to the study of biological heart valve roots.

#### Development of the real time wear system

**Fig 4:**
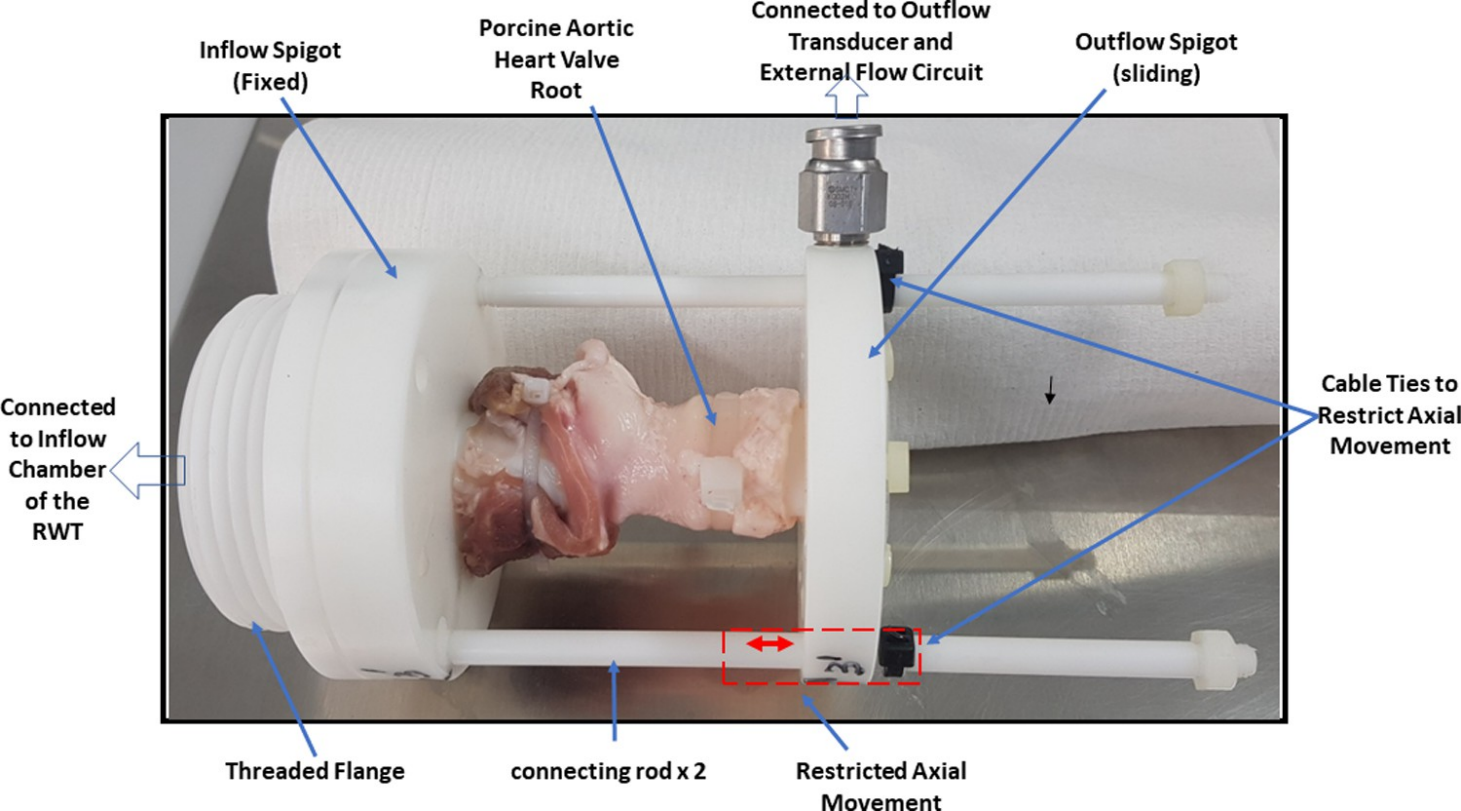
Porcine aortic heart valve root mounted on the bespoke heart valve root holder with restricted axial movement of the outflow spigot.

In order for the RWT to be used with biological heart valve roots, a holder was designed and manufactured to accommodate the porcine aortic heart valve root. The heart valve root holder design comprised of two spigots (inflow and outflow), connected and supported by two connecting rods allowing axial and circumferential expansion of the heart valve root wall due to the pulsatile flow (Fig 4). The inflow spigot connected directly to the threaded flange from the original valve holder for the RWT; the outflow spigot slid axially on the two connecting rods, which contained end stops to restrict axial movement to the range required and prevent detachment (Fig 4). Additionally, the outflow spigot had a push fitting to attach a digital pressure transducer to record outflow pressure (pressure in the aorta).

**Fig 5:**
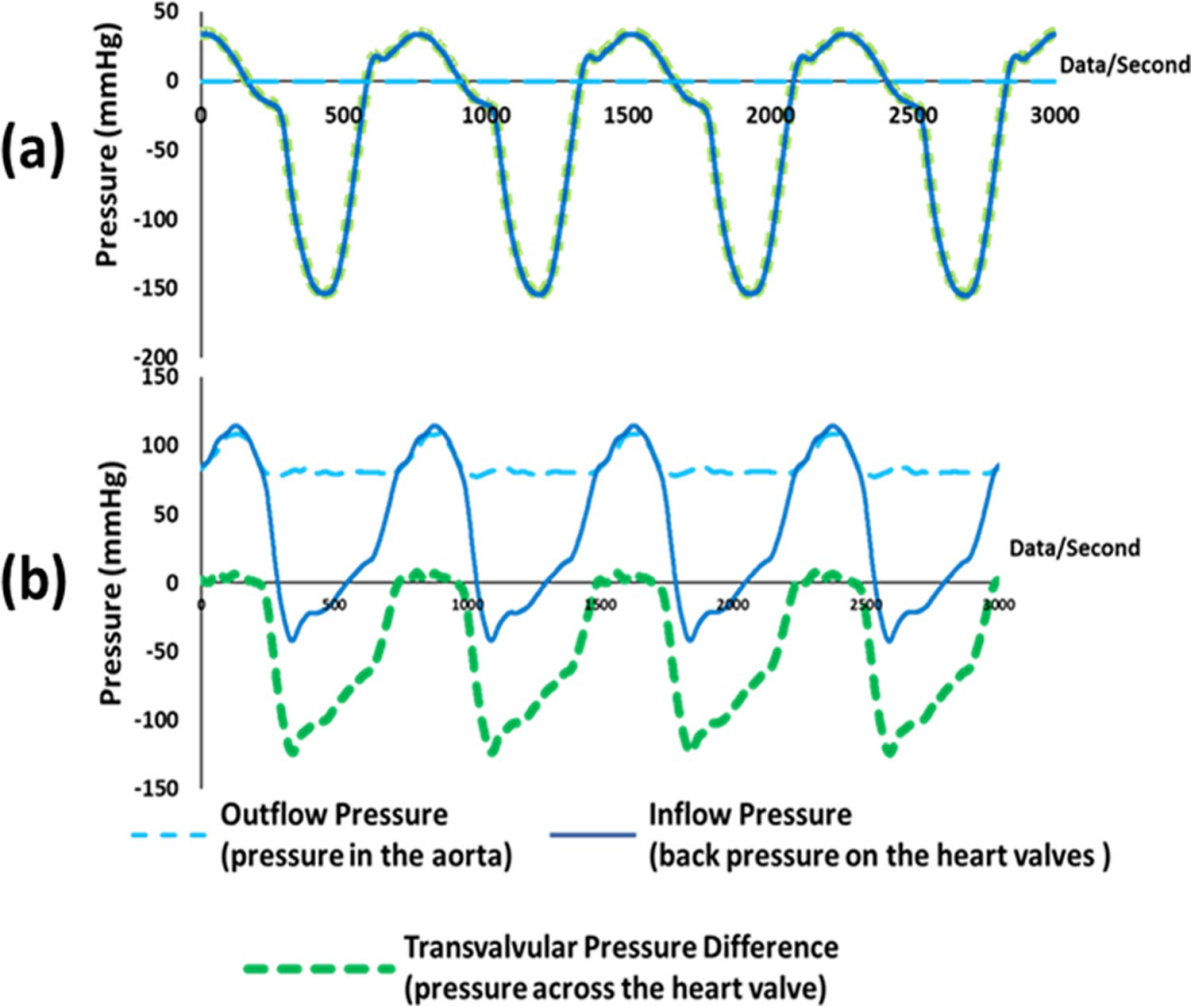
Typical pressure waveforms at heart rate 120 bpm during real time fatigue assessment of porcine aortic heart valve roots in the RWT (a) without any modifications (b) with modification of the RWT to maintain physiological contraction-expansion movement of the aorta.

Fig 5(A) shows typical pressure waveforms for the porcine aortic heart valve roots without modification to the RWT; here the negative inflow pressure waveform was due to the pump piston backward stroke and the zero outflow pressure was due to the outflow chamber being open to the atmosphere. This arrangement affected the wall of the heart valve root, resulting in the wall collapsing and hence non- physiological flow through the valve. Hence, in order to keep the aorta dilated and maintain an appropriate physiological contraction-expansion movement, the outflow spigot was connected to a newly developed external flow circuit containing a test solution that applied pressure within the heart valve root using a vertical pressure column (compliance chamber). This assembly generated pressure in the aorta of the heart valve root as shown by the positive outflow mean pressure range of 92 ± 16 mmHg in Fig 5(B), ensuring the root did not collapse and also serving as a fluid reservoir. The full modified arrangement can be seen in Fig 2, which features three porcine aortic heart valve roots mounted in the modified RWT.

#### Real time mechanical fatigue assessment test conditions

Three heart valve roots were studied simultaneously in the modified RWT described above using a sinusoidal pulsatile flow input waveform at a physiological heart rate of 120 bpm for up to 1.2 million cycles. The three remaining stations were blanked off. The test solution used was 0.9% (w/v) saline supplemented with 0.03% (v/v) sodium azide to retard microbial growth. To measure back pressure on the test valves, the inflow pressure transducer was mounted on top of the inflow chamber and to measure pressure in the aorta, the outflow pressure transducer was mounted on the outflow spigot of one station as the pressure was found to be similar in each station during validation testing. To achieve full valve closing and opening and simultaneously controlling mean transvalvular pressure across the closed valve, the amplitude of the input waveform and the flow bypass valve setting was adjusted systematically until the desired conditions were achieved. A mean transvalvular pressure difference of -100 mmHg across the closed valves for over 95% of the test cycles and for at least 5% of each cycle duration in accordance with ISO 5840 [37] was used as the input condition. The actual outflow pressure (aorta pressure) was maintained between 92 ± 16 mmHg. During the study, the heart valve roots were checked daily for any artery leakage and alteration of transvalvular pressure difference across the closed valve. At the end of the study, the real time mechanical fatigue of heart valve root leaflets was assessed in the RWT by capturing images of the leaflet dynamics at 500 frames.s^-1^ using a high-speed camera (S-PRI Model, AOS Technologies, Inc) and through qualitative visual assessment.

#### Competency (pre-test, mid-test, post-test) assessment

The competency of each heart valve root was determined in terms of static leakage flow rate at pre-defined intervals (pre-test, mid-test and post-test) of the fatigue assessment. The leakage flow rate was obtained by applying a static back pressure on the valve root with a column of 0.9% (w/v) saline. The time taken for the pressure of the test fluid to drop from 120 to 80 mmHg was recorded and the leakage flow rate calculated [19]. The heart valve root was classified as competent if the pressure head had not dropped to 80 mmHg within a cut off time period of 30 min, i.e. the leakage flow rate was ≤ 0.85 mL.s^-1^.

#### Pulsatile flow assessment

The hydrodynamic performance of all the heart valve roots was determined in an optimised Leeds pulsatile flow simulator [38, 39] following real time mechanical fatigue and post-test competency assessment. A viscoelastic impedance adapter (VIA; Vivitro System Inc., Victoria BC, Canada) was used to deliver a more physiological ventricle pressure and flow waveform in the simulator. All the heart valve roots were initially tested at minimum compliance in the pulsatile flow simulator with heart rates of 60, 72, 80 and 100 bpm with corresponding stroke volumes of 60, 70, 70 and 80 ml. The systemic pressure was held between 120 and 80 mmHg. Each test was repeated with the VIA adjusted to its maximum compliance setting to produce relevant physiological pressure and flow characteristics [39].

**Fig 6:**
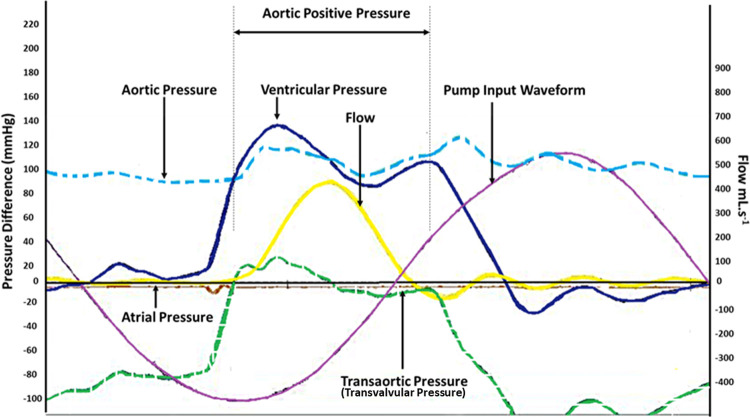
Typical pressure and flow waveforms at heart rate 72 bpm under maximum compliance during pulsatile flow assessment of cellular and decellularised porcine aortic heart valve roots in the Leeds pulsatile flow simulator.

As shown in Fig 6, the pump input waveform was a sine wave. Pressure measurements were taken at three positions in the simulator; upstream of the aortic root (ventricle pressure in Fig 6), downstream of the aortic root (aortic pressure in Fig 6), and in the filling chamber (atrial pressure in Fig 6). The first two allowed the pressure difference across the aortic root to be determined (transaortic pressure in Fig 6). An electromagnetic flowmeter measured the flow downstream of the aortic root.

To assess the pulsatile performance of the heart valve roots, transvalvular pressure difference during forward flow (ΔP), the root mean square (RMS) forward flow (Q_RMS_), effective orifice area (EOA) and leaflet dynamics were evaluated. The EOA was evaluated using the formula EOA = Q_RMS_ (mL.s^-1^)/51.6√ΔP (mmHg) [40]. The EOA was presented as the mean ± 95% confidence limits. The leaflet dynamics were recorded with an AOS Technologies S-PRI high-speed camera at 500 frames.s^-1^ at the maximum compliance condition at the 72 bpm heart rate condition. All tests were performed using physiological 0.9% (w/v) saline. Regurgitant volumes could be acquired from pulsatile flow assessment; however these were considered unreliable due to artefactual flow oscillations in the heart valve root during diastole and hence were not included in the outcomes.

#### Uniaxial tensile properties of leaflets

**Fig 7:**
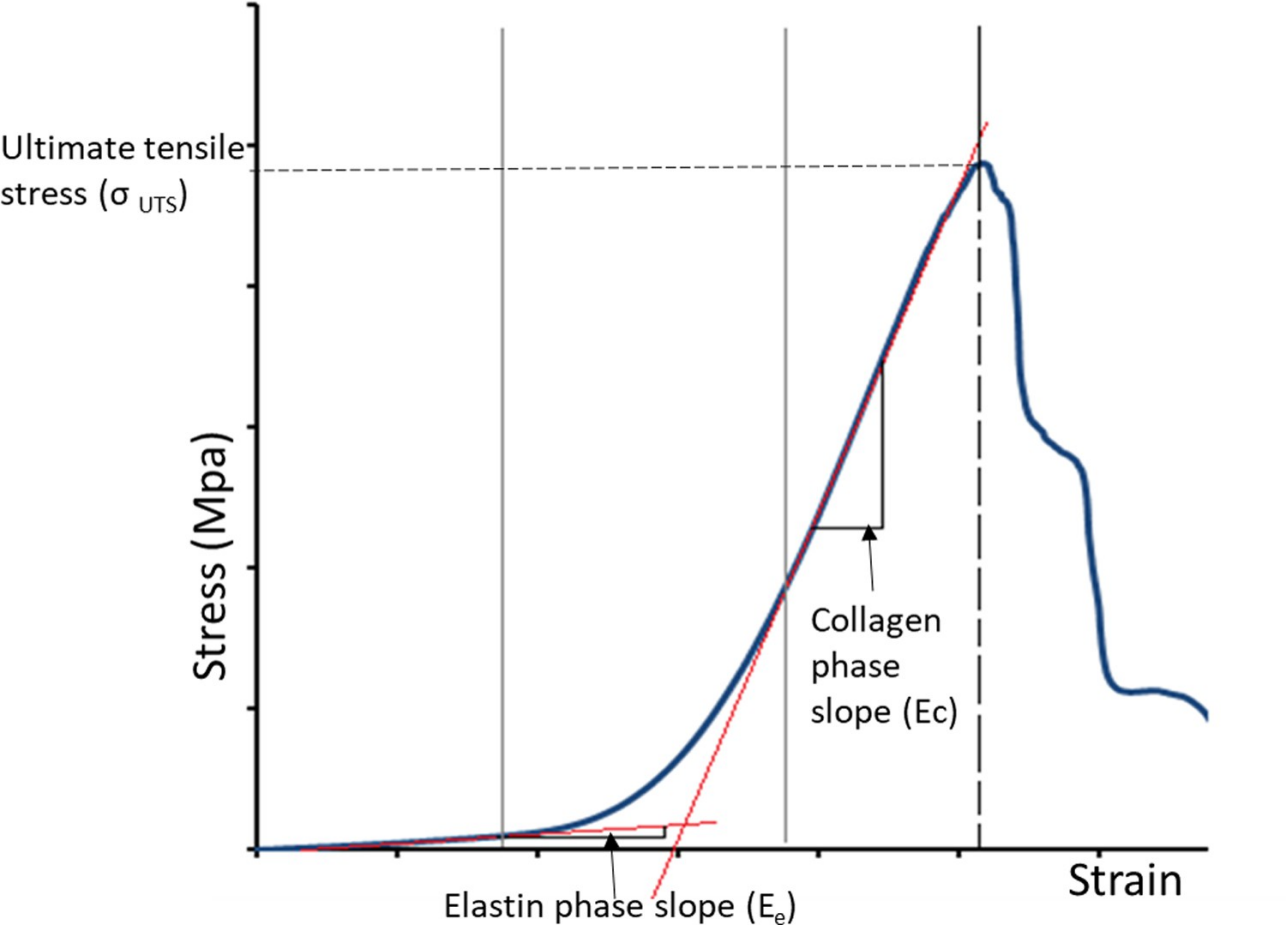
Typical stress-strain graph of a heart valve leaflet specimen subjected to uniaxial tensile loading to failure.

The effect of cyclic loading on the material properties of the heart valve leaflets was characterised using uniaxial tensile testing in both the circumferential and radial directions, following competency, fatigue and pulsatile flow assessment. Heart valve leaflet specimens measuring 5 × 10 mm in the circumferential and 3 x 6 mm in the radial directions were dissected from each heart valve root and subjected to uniaxial tensile loading to failure using an Instron 3365 (Instron, Bucks, UK). Prior to testing, the thickness of the specimens was measured six times along the gauge length using a thickness gauge with a resolution of 0.01 mm (J-40-V; James H. Heal and Company Limited), and their average thickness was calculated. Subsequently, the specimens were mounted onto a purpose-built holder and stretched to failure at a rate of 10 mm·min^−1^ similar to that previously reported in studies of human aortic and pulmonary [19] and porcine pulmonary [9] heart valve roots. The recorded load and extension data were converted to engineering stress and engineering strain. The stress–strain behaviour of each leaflet specimen was characterised by means of three biomechanical parameters: elastin phase slope (E_e_), collagen phase slope (E_c_) and ultimate tensile stress (σ _UTS_) as previously described by Hasan, Ragaert [41]. Elastin phase slope and collagen phase were created through linear regression of the linear portions of the stress–strain graph (Fig 7), performed in Microsoft Excel. In order for a test to be valid the trend line was required to possess an R^2^ > 0.85. The maximum stress value was recorded as the Ultimate Tensile Strength (UTS).

#### Statistical analysis

All the hydrodynamic and biomechanical performance numerical data for the aortic heart valve roots were analysed using Microsoft Excel 2013 and presented as the mean ± 95% confidence limit. Statistical significance between the cellular and decellularised porcine aortic heart valve roots hydrodynamic and biomechanical performance was determined using Student’s t-test. A significance level of p < 0.05 was applied. Statistical analyses were performed using SPSS for Windows (version 21.0; SPSS, Inc., USA).

## Results

### Real time fatigue of porcine aortic heart valve roots

**Fig 8:**
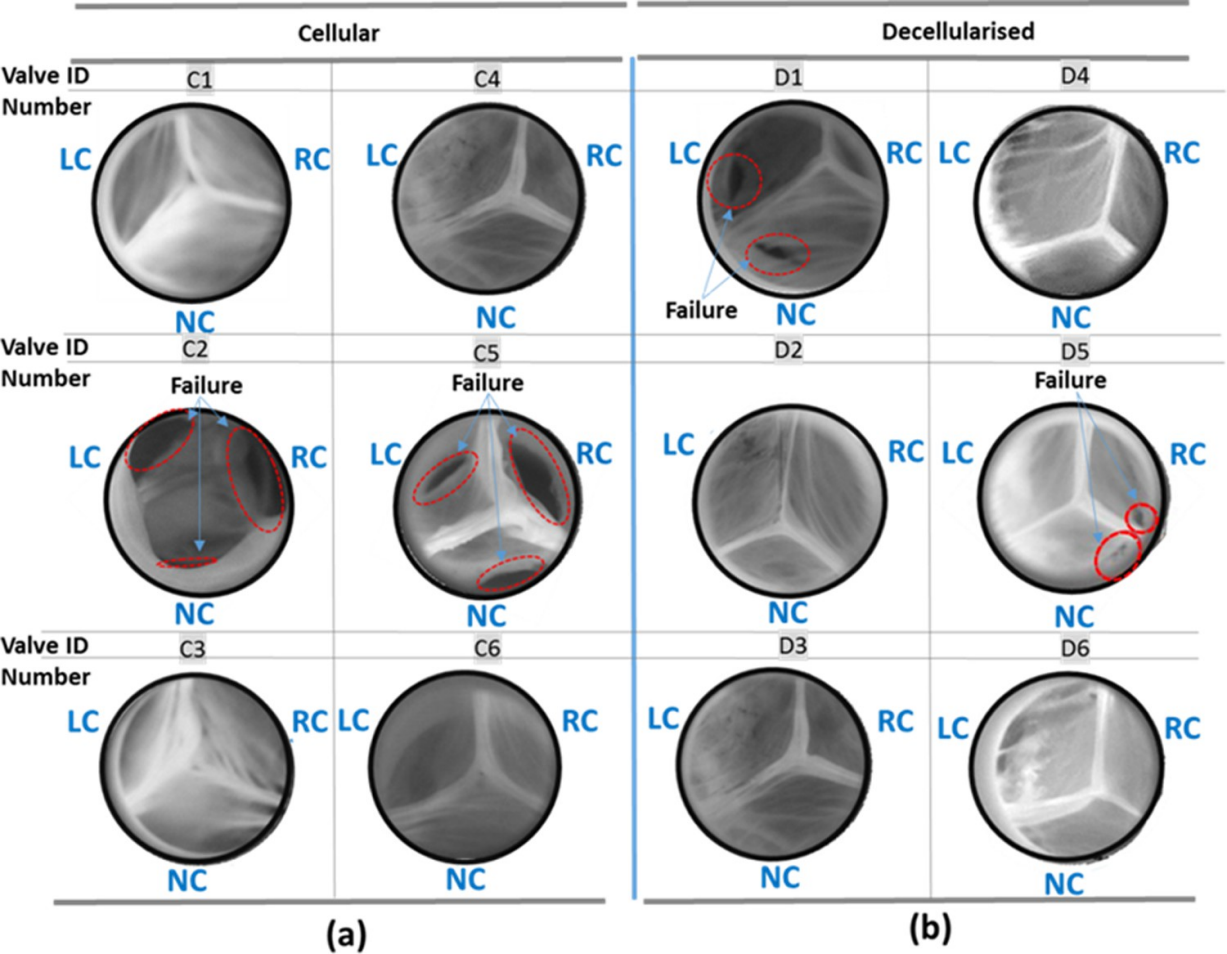
Valve fully closed images (where LC: left coronary leaflet, RC: right coronary leaflet and NC: non-coronary leaflet) of the (a) cellular (b) decellularised porcine aortic heart valve roots at maximum 1.2 million cycles with leaflet tears circled in red, captured during real time fatigue assessment.

A novel *in vitro* real time mechanical fatigue assessment method and procedure was developed by modifying a real time wear tester (RWT). The procedure that was developed permitted a mean transvalvular pressure difference (100 mmHg) across the closed cellular and decellularised heart valve roots to be maintained (actual pressure difference 92 ± 16 mmHg) Further, there was no sign of detachment of the heart valve roots from the mounting arrangement that was developed. The procedure developed allowed testing for 1.2 million cycles over 10 days. A small amount of yellow staining on the cellular heart valve root walls was observed. In situ high speed camera images of the fully closed position for cellular and decellularised porcine aortic heart valve roots at the end of the real time fatigue assessment are shown in Fig 8.

For both the cellular and decellularised heart valve root experimental groups, four of the six aortic heart valve roots (decellularised: D2, D3, D4 and D6; cellular: C1, C3, C4 and C6) were cycled to maximum 1.2 million cycles without signs of fatigue failure, whereas two heart valve roots from each experimental group (decellularised: D1 and D5; cellular: C2 and C5) exhibited tears near to the belly region of the leaflets (Fig 8).

### Competency of porcine aortic heart valve roots

The post-test competency of two of the cellular (C3 and C5) and one of the decellularised (D1) and mid competency of one of the decellularised (D1) aortic heart valve roots could not be measured as the valves were leaking faster than the static pressure could be applied.

The pre, mid and post-test mean static leakage flow rates for the cellular and decellularised aortic heart valve roots are listed in Table 1.

**Table 1:** Pre, mid and post- test mean static leakage flow rates of each cellular and decellularised porcine aortic heart valve root.

Cellular Aortic Heart Valve Roots				Decellularised Aortic Heart Valve Roots			
Valve ID Number	Leakage Flow Rate (mL.s^-1^)			Valve ID Number	Leakage Flow Rate (mL.s^-1^)		
	Pre-test	Mid-test	Post-test		Pre-test	Mid-test	Post-test
C1	<0.85	<0.85	4.36	D1	2.05	xxx	xxx
C2	<0.85	<0.85	1.53	D2	0.88	0.99	<0.85
C3	<0.85	<0.85	xxx	D3	1.61	4.73	6.02
C4	<0.85	<0.85	1.19	D4	<0.85	<0.85	<0.85
C5	<0.85	<0.85	xxx	D5	2.24	21.31	1.23
C6	<0.85	16.67	9.96	D6	1.64	1.23	1.66

xxx—not measured

All the cellular aortic heart valve roots (C1, C2, C3, C4, C5 and C6) were fully competent pre- test, with mean leakage flow rates of less than 0.85 mL.s^-1^. Post-test, none of the six cellular aortic heart valve roots were considered fully competent as they all had leakage rates higher than 0.85 mL.s^-1^.

For the decellularised aortic heart valve roots, pre-test only one of these was considered fully competent (D4), although root D2 was almost classed as fully competent. Post-test, these two (D2, D4) roots were still fully competent.

### Pulsatile flow performance of porcine aortic heart valve roots

The pulsatile flow performance of two of the cellular porcine aortic heart valve roots (C2 and C5) could not be determined as all the leaflets were too damaged for testing to be completed. In addition, it was not possible to assess the pulsatile flow performance of two of the cellular porcine aortic heart valve roots (C1 and C6) at the heart rate of 100 bpm as the myocardium side of the heart valve root detached from the spigot when this condition was applied.

**Fig 9:**
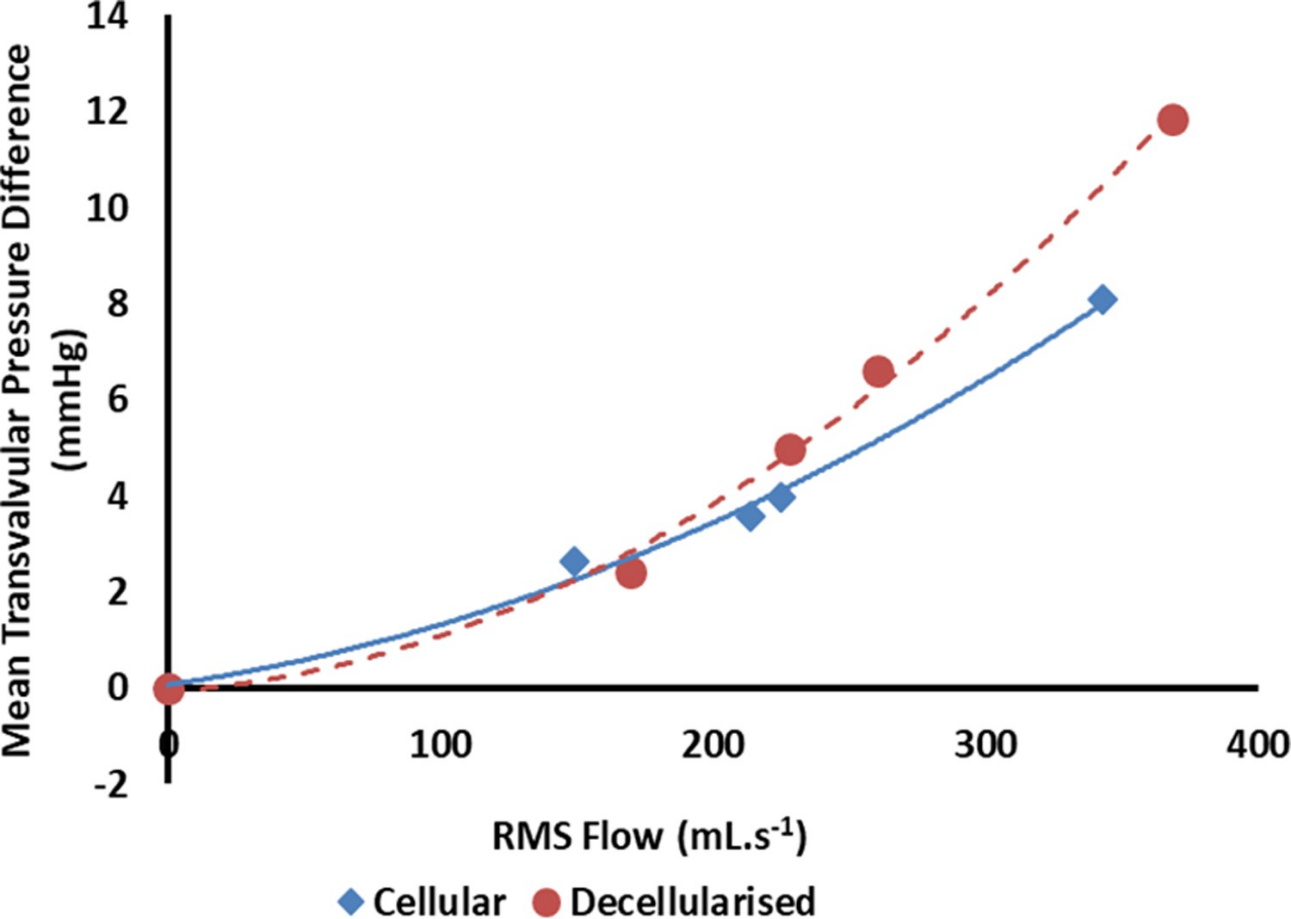
Mean transvalvular pressure difference versus mean RMS flow for cellular (n = 4) and decellularised (n = 6) porcine aortic heart valve roots–data fitted with second order polynomial trend line.

The mean transvalvular pressure difference with respect to RMS flow across the decellularised aortic heart valve roots showed a similar trend to the cellular aortic heart valve roots (Fig 9) and the mean EOA for the decellularised porcine aortic valve heart valve roots (2.06 ± 0.10 cm^2^) was not significantly different to the cellular porcine aortic heart valve roots (2.23 ± 0.21 cm^2^) (P = 0.08, Student’s t-test).

**Fig 10:**
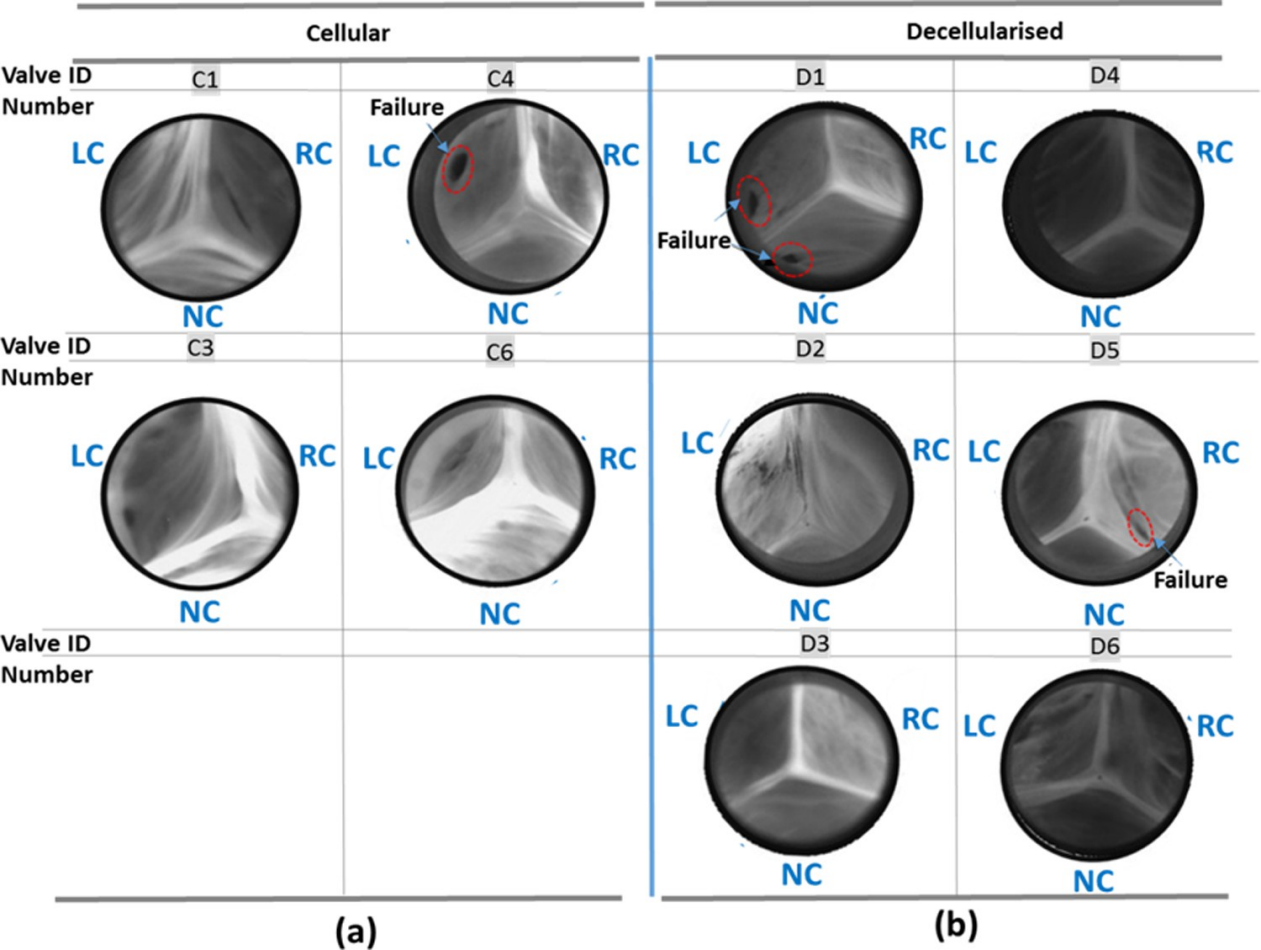
Valve fully closed images of (a) cellular (b) decellularised porcine aortic heart valve roots at heart rate 72 bpm during pulsatile flow assessment, captured from the front view by a high-speed camera with leaflet tears circled in red.

Representative images of the cellular and decellularised valves fully closed during a cardiac cycle with heart rate 72 bpm are shown in Fig 10.

For three of four cellular aortic heart valve roots (C1, C3 and C4), the leaflets opened fully, producing circular or near-circular orifices. However, in one of the cellular aortic heart valve roots (C6), the leaflets did not fully open resulting in a restricted triangular orifice. All four cellular aortic heart valve roots tested appeared competent since a central leakage orifice was negligible or not observed during the closed phase however, a large tear was detected near the leaflet attachment area of the left coronary leaflet of one sample (C4) which was not seen in the post-test fatigue image analysis assessment in the RWT (Fig 8).

The majority of the decellularised porcine aortic heart valve roots showed synchronous leaflet opening and closing characteristics, except in one of the decellularised heart valve roots (D5), the right coronary leaflet did not open completely. The fully closed leaflet configuration was excellent for four of the decellularised aortic heart valves (with no visible central leakage orifice) and a minor central leakage orifice was observed in the two remaining decellularised aortic heart valve roots.

### Uniaxial tensile properties of aortic leaflets

It was not possible to determine the tensile properties of the leaflets from two of the cellular aortic heart valve roots because the leaflets were torn during the cyclic fatigue and pulsatile flow study and it was not possible to prepare an adequate size of specimen for the uniaxial tensile testing. In addition, one radial and one circumferential decellularised leaflet specimens were excluded from the analysis for E_e_ because the initial linear region of the stress-strain curve used to derive the elastin phase modulus was not distinct. Hence, these samples were omitted from the analysis and five decellularised samples were used for the mean elastin phase slope calculation in each direction.

The biomechanical parameters of the decellularised and cellular aortic heart valve root leaflets are listed in Table 2. A comparison between the decellularised and cellular porcine aortic heart valve root leaflets revealed no significant difference between the leaflet parameters in the radial direction (p>0.05, Student’s t-test). A significant increase in the elastin phase modulus, collagen phase modulus and UTS in the circumferential direction was observed in the decellularised aortic leaflet specimens compared to the cellular leaflet specimens (p<0.05, Student’s t-test).

**Table 2:** Tensile parameters (collagen phase slope Ec, elastin phase slope Ee, ultimate tensile stress σ _UTS_) ± 95% confidence limits for cellular and decellularised radial and circumferential aortic leaflet specimens and number of test specimens (N) *-Statistical significant difference (p<0.05) for decellularised versus cellular aortic heart valve roots.

Porcine Aortic Heart Valve Roots					
Tensile Parameters for Leaflets	Cellular		Decellularised		p value
	N		N		
**Radial**					
**E**_**c**_ **(MPa)**	4	0.93 ± 0.98	6	2.47 ± 1.60	0.10
**E**_**e**_ **(MPa)**	4	0.02 ± 0.01	5	0.03 ± 0.01	0.07
**σ**_**UTS**_ **(MPa)**	4	0.46 ± 0.36	6	1.06 ± 0.68	0.11
**Circumferential**					
**E**_**c**_ **(MPa)**	4	8.59 ± 7.92	6	53.25 ± 8.99*	<0.01
**E**_**e**_ **(MPa)**	4	0.02 ± 0.01	5	0.31 ± 0.25*	0.02
**σ**_**UTS**_ **(MPa)**	4	2.48 ± 0.66	6	9.31 ± 1.65*	<0.013

The data associated with this paper is available from the University of Leeds Data Repository [42].

## Discussion

A critical step in translation of decellularised heart valve roots for use as replacement heart valves, is to have better understanding of *in vitro* durability and failure mechanisms due to the impact of cyclic physiological loading. Many studies [24, 26, 27, 43–48] have reported the fatigue and durability of replacement heart valves under accelerated cyclic loading conditions. The fatigue of conventional mechanical and bioprosthetic (glutaraldehyde fixed) replacement heart valves have been evaluated using the accelerated fatigue assessment method and showed similar fatigue in comparison to the fatigue of retrieved *in vivo* heart valves [47, 48]. However, for heart valves comprising of leaflets with a greater visco-elastic response, such as polymer materials and (non-glutaraldehyde fixed) biological tissue the fatigue mechanism was not predictive of *in vivo* wear mechanisms [24–26]. Butterfield and Fisher [26] explained that during accelerated fatigue assessment, valve leaflets do not have enough relaxation time and experience additional non physiological forces. Furthermore, other failure modes may occur in real time associated with creep or incremental accumulation of plastic deformation, which may not be replicated in accelerated testing. Therefore fatigue assessment of biological heart valve roots needs to be performed under physiological loading conditions.

In this preliminary study, a novel *in vitro* real time mechanical fatigue assessment method for biological heart valve roots has been developed. This was achieved through the modification of a RWT system, through the development of a holder to accommodate heart valve roots, and modification of the system by adding a compliance chamber to achieve more physiological conditions. With the addition of the compliance chamber, resistance to the piston increased and hence reduced the negative inflow back pressure on the valve as shown in Fig 5(B), maintaining contraction-expansion movement of the aortic root wall. This contraction-expansion movement of the aortic root wall was dependent on the height of the fluid in the compliance chamber. The real time fatigue assessment of cellular and SDS decellularised porcine aortic heart valve roots showed leaflet tears in both decellularised and cellular porcine aortic heart valve roots. This leaflet structural failure could either have been due to leaflet impingement with the heart valve root mounting spigot of the heart valve root holder (Fig 4), or fatigue induced deterioration. At this point in the research study it cannot be determined exactly what caused the leaflet failure. However, this study emphasised that arterial compliance was an important factor in recreating more physiological pressure conditions. The arterial compliance in aortic heart valve roots is assumed to prevent the leaflets from sticking to the wall of the aorta [49] and to transmit the stress from the leaflets to the wall [50]; and help to reduce leaflet stresses and thereby fatigue and eventual structural failure of the valve leaflets [51]. In addition, the *in vitro* RWT setup accelerated cellular death (necrosis) within a few hours of testing in the cellular aortic heart valve roots due to (toxic) sodium azide which was added in the test solution [0.9% (w/v) saline with 0.04% sodium azide (v/v)] to retard bacterial growth, and the lack of oxygen supply (hypoxia); therefore, only the matrix material was tested in the RWT. Necrosis is characterised by the release of hydrolytic enzymes (including proteinases), that are stored by lysosomes, which are capable of digesting cell components or the entire cell itself [52]. Excessive amounts of these enzymes could damage extracellular matrix proteins (collagen) surrounding the cells which form a highly organised fibrous structure in the cellular heart valve root leaflets. In addition, cell membranes in cellular heart valve roots may have been damaged soon after harvesting due to handling and processing [53]. Hence cell necrosis may have contributed to the mechanical damage and tears of the cellular heart valve leaflets. A small amount of yellow staining was observed on the cellular heart valve roots walls possibly due to the growth of bacteria/fungi and nature of unsterile testing. By treating the cellular porcine aortic heart valve roots with Cambridge antibiotic solution and amphotericin B, this may have helped in minimising the growth of contaminating bacteria and fungi.

However, cell necrosis would not explain the fatigue damage observed in the decellularised porcine aortic roots, which were devoid of cells and exhibited similar fatigue damage (tears in the leaflets) as the cellular heart valve roots. Although what each experimental group did have in common was that neither type had any living cells present to help "repair" the damaged collagen that would occur under physiological conditions *in vivo* in the heart. Once the decellularised heart valve root is implanted into the human body for use as a replacement heart valve, it is believed that leaflet fatigue will not be permanent due to cellular population of the leaflet and repair. *In vivo*, it is anticipated, based on evidence from a study [11] on decellularised porcine aortic valve roots implanted in sheep, that host cells will populate the leaflets and remodel the matrix.

In summary, the mechanical fatigue damage in the cellular and decellularised heart valve roots appeared to be similar in nature but may have occurred due to a combination of various factors such as leaflet impingement with the spigot of the heart valve root holder, cell necrosis or fatigue induced leaflet deterioration during fatigue assessment in the RWT.

Failure of the leaflets to co-apt properly in diastole due to heart valve root dilation or stiffening of the leaflets ultimately can cause aortic regurgitation [54]. Therefore the competency assessment of heart valve roots at several intervals during the fatigue study was performed to assess the effect of repeated cyclic loading on valve closure. Periodical competency assessment results showed that regurgitation was variable from valve to valve in both cellular and decellularised heart valve roots and cyclic fatigue did not adversely affect valve leaflet closure and thereby regurgitation on the valve competency (static leaflet closure).

The decellularised porcine aortic heart valve roots showed comparative functionality to the cellular heart valve roots under *in vitro* pulsatile flow conditions in terms of EOA, transvalvular pressure difference and leaflet kinematics. Clinically the functional parameters such as EOA and transvalvular pressure difference are important indicators for the clinical assessment of valve stenosis severity [55, 56].

The biomechanical tensile properties of the leaflets showed no significant differences between cellular and decellularised leaflet parameters in the radial direction. This leads to the conclusion that the physiological cyclic loading did not alter radial mechanical tensile properties of the cellular and decellularised leaflets. The response in the radial direction is dominated by elastin fibres within the leaflets, which provide resistance to large radial strains when the valve is fully open. Moreover, it is noteworthy that the decellularised aortic leaflets were significantly stronger and stiffer in the circumferential direction compared to the cellular aortic leaflets. The circumferential characteristics of the leaflets are dominated by load bearing collagen fibres which provide strength to maintain leaflet coaptation during diastole. Such changes may be due to the degeneration of cellular leaflets in the RWT. The tensile parameters in this study were lower than the tensile parameters derived by Desai [57] for cellular porcine aortic leaflets, indicating degeneration of the cellular aortic leaflets. However, this was not the case for the decellularised aortic leaflets as the collagen and elastin phase slopes for decellularised aortic leaflets were not only higher than those of the cellular aortic leaflets post-fatigue, but also higher than the collagen and elastin phase slopes derived by Korossis [58] on porcine aortic heart valve roots decellularised with a similar protocol but that had not undergone fatigue loading. This showed that the decellularised aortic leaflets appeared to maintain their overall structural integrity.

### Limitations of the study

The study was based on a relatively small sample size (n = 6) which meant a lack of statistical power to discriminate/provide significant differences between mechanical fatigue of the cellular and decellularised heart valve roots.

Fundamentally the RWT has predominantly been designed to the meet the requirements of ‘Durability Testing’ within ISO5840-1 (2021), which includes maintaining a peak transvalvular pressure of 100 mmHg across the closed valves (for at least 5% of each cycle), and allows the longer term (fatigue) performance of multiple valves to be determined simultaneously. Due to this, there are a number of limitations in terms of hydrodynamics of the system, especially when compared to a more conventional pulsatile flow simulator. For example, a pulsatile flow simulator (such as the one used in this study for the hydrodynamic assessment) consists of a circular flow loop, whereas the RWT did not, simply using a pump piston mechanism to displace fluid forwards and backwards through the valve, generating pressure. Crucially, the main difference between the RWT and pulsatile flow simulator is the (ventricular) pressure waveforms during valve closure. In the unmodified RWT the inflow pressure decreases slowly with an almost linear decrease to zero and turns negative since the piston is still moving backward even after valve closure [Fig 5(A)]. Whereas in the pulsatile flow simulator the ventricular pressure waveform is close to zero during the majority of the diastolic phase (Fig 6). This limitation was mitigated by the inclusion of a vertical pressure column/compliance chamber, which reduced the negative pressure.

Further, whereas the input conditions of the pulsatile flow simulator include stroke volume and peripheral resistance, the only controllable input condition (aside from frequency, controllable in both types of system) for the RWT was the applied pressure on valve closure, controlled by the bypass valve mechanism. Finally, due to the absence of a flow meter in the RWT system, pressure measurements only could be obtained and analysed.

There was no consideration of the effects of tissue remodelling due to cell repopulation and structural valve degeneration due to calcification which may influence the valve fatigue, leaflet mechanical properties and flow characteristics of the decellularised heart valve roots. Tissue remodelling will play an important role in the durability of decellularised heart valve roots, while enzymatic degradation and calcification will limit their durability and fatigue. Also short term fatigue assessment due to lack of cellular tissue viability and regeneration of heart valve roots was a limitation of this work. Therefore direct comparison of *in vitro* fatigue with *in vivo* clinical data may be inappropriate. However, the more physiological loading rate is an improvement over the previous accelerated durability studies. Future work will compare this real time fatigue assessment method with accelerated fatigue assessment method.

## Conclusion

In conclusion, a novel physiological *in vitro* real time fatigue assessment method that can predict mechanical fatigue of biological heart valve roots has been developed in this preliminary study. This method could be used as one of a portfolio of *in vitro* preclinical assessment tools to better predict the efficacy and reliability of new heart valve replacements at early stage, even before embarking on *in vivo* animal trials (and thereby reducing animal sacrifice and cost), particularly for those that possess visco-elastic properties. The *in vitro* real time fatigue of cellular and SDS decellularised porcine aortic heart valve roots was evaluated in this study for the first time. The majority of decellularised porcine aortic heart valve roots not only resisted physiological cyclic loading up to 1.2 million cycles but also maintained their hydrodynamic function and leaflet mechanical properties.
